# Association of anti-diabetic drugs and covid-19 outcomes in patients with diabetes mellitus type 2 and chronic kidney disease: Nationwide registry analysis

**DOI:** 10.1371/journal.pone.0301056

**Published:** 2024-03-27

**Authors:** Jelena Dimnjaković, Tamara Buble, Pero Ivanko, Tamara Poljičanin, Sandra Karanović Štambuk, Hana Brborović, Ognjen Brborović

**Affiliations:** 1 Division for Health Informatics and Biostatistics, Department for Biostatistics, Croatian Institute of Public Health, Zagreb, Croatia; 2 Health Center "Dom zdravlja Zagreb zapad", Zagreb, Croatia; 3 Department of Nephrology, Arterial Hypertension, Dialysis and Transplantation, University Hospital Centre Zagreb, Zagreb, Croatia; 4 Department of Internal Medicine, School of Medicine, University of Zagreb, Zagreb, Croatia; 5 Department of Environmental and Occupational Health and Sports Medicine, School of Medicine, University of Zagreb, Zagreb, Croatia; 6 Department of Social Medicine and Organization of Health Care, School of Medicine, University of Zagreb, Zagreb, Croatia; Pelita Harapan University Faculty of Medicine: Universitas Pelita Harapan Fakultas Kedokteran, INDONESIA

## Abstract

**Introduction:**

Patients with diabetes mellitus type 2 and chronic kidney disease (T2DM-CKD) have a 5 times higher risk of developing severe SARS-CoV-2 infection than those without these 2 diseases. The goal of this study is to provide information on T2DM-CKD and COVID-19 outcomes, with an emphasis on the association with anti-diabetic medications.

**Methodology:**

Study is designed as a retrospective cohort analysis covering the years 2020 and 2021. Data from the National Diabetes Registry (CroDiab) were linked to hospital data, primary healthcare data, Causes of Death Registry data, the SARS-CoV-2 vaccination database, and the SARS-CoV-2 test results database. Study outcomes were cumulative incidence of SARS-CoV-2 positivity, COVID-19 hospitalizations, and COVID-19 deaths. For outcome predictors, logistic regression models were developed.

**Results:**

Of 231 796 patients with diabetes mellitus type 2 in the database, 7 539 were T2DM-CKD (3.25%). The 2-year cumulative incidences of all three studies’ outcomes were higher in T2DM-CKD than in diabetes patients without CKD (positivity 18.1% vs. 14.4%; hospitalization 9.7% vs. 4.2%; death 3.3% vs. 1.1%, all p<0.001). For COVID-19 hospitalization, protective factors were SGLT-2 inhibitors use (OR 0.430; 95%CI 0.257–0.719) and metformin use (OR 0.769; 95% CI 0.643–0.920), risk factors were insulin use (1.411; 95%CI 1.167–1.706) and sulfonylureas use (OR 1.226; 95% CI 1.027–1.464). For SARS-CoV-2 positivity protective factors were SGLT-2 inhibitors (0.607; 95% CI 0.448–0.823), repaglinide use (OR 0.765; 95% CI 0.593–0.986) and metformin use (OR 0.857; 95% CI 0.770–0.994). DPP-4 inhibitors showed a non-significant decrease in risk for COVID-19 death (OR 0.761; 95% CI 0.568–1.019).

**Conclusion:**

T2DM-CKD are heavily burdened by COVID-19 disease. Our results suggest no association between antidiabetic drugs and COVID-19 death outcome while SGLT-2 and metformin show to be protective against COVID-19 hospitalization and infection, repaglinide against infection, and insulin and sulfonylureas show to be risk factors for COVID-19 hospitalization and infection. Further research in T2DM-CKD is needed.

## Introduction

At the moment of this paper writing, COVID-19 is no longer defined as a Public Health Emergency of International Concern. However, it continues to take a significant toll on health globally due to the continued widespread circulation of the virus [1–3]. Also, it is estimated that at least 17 million people experienced Post COVID-19 Condition in the first 2 years of the pandemic and that number potentially doubled to over 34 million in 2022 [2].

Very early in the course of pandemics, it was clear that patients with comorbidities are in greater danger from developing more severe forms of COVID-19 disease than the otherwise healthy people [4–14]. This is especially true for patients with diabetes and patients with CKD [15, 16].

However, multimorbidity, the presence of two or more long-term health conditions, a major growing public-health challenge, makes the person even more prone to dying from COVID-19 [15, 17]. This is especially true for patients with diabetes mellitus type 2 and chronic kidney disease (T2DM-CKD) who have up to 7.22 times higher odds of severe infection in comparison to people without these 2 diseases [18].

Another topic that has been interesting to the scientific community and among clinicians is the effect of antidiabetic drugs on COVID-19 outcomes [19]. There were uncertainties if these drugs might lead to worse outcomes of COVID-19 in patients with diabetes [5, 19–28]. For the population of T2DM-CKD, antidiabetics with renoprotective effect and their association with COVID-19 outcomes might be of special interest—SGLT-2 inhibitors and GLP-1 analogues [15, 28].

Scientific activity analyzing associations of antidiabetics and COVID-19 outcomes in DM2 patients is live although no firm conclusions have been made yet. On the other hand, we found no studies conducted specifically on the heavily burdened T2DM-CKD population.

In our study, our goals were to:

1. Evaluate the prevalence of T2DM-CKD,
2. Evaluate the 2-year cumulative incidences (years 2020 and 2021) of SARS-CoV-2 infections, COVID-19 hospitalizations, and COVID-19 deaths among T2DM-CKD,
3. Analyze risk factors for SARS-CoV-2 infections, COVID-19 hospitalizations, and COVID-19 deaths in the observed population with a focus on anti-diabetic therapy.

## Methodology

The study was a retrospective data analysis covering the period from, Jan 1^st^, 2020 to Dec 31^st^, 2021. Characteristics of the entire population of patients with diabetes mellitus type 2 in Croatia were analyzed, focusing on the sub-population of people with chronic kidney disease (T2DM-CKD).

Croatian National Diabetes Registry (CroDiab) was the source of data. CroDiab contains individual longitudinal data on patients with diabetes mellitus [29, 30]. Several sources are being used to feed CroDiab with data via the National Public Health Information System of Croatia and the Central Health Information System of the Republic of Croatia: clinical laboratories, primary health care providers, and hospitals [31, 32]. For our study, CroDiab was linked to a database containing SARS-CoV-2 test results, the National Vaccination Database (eVac), and the National Causes of Death Registry using a common personal identifier [33, 34]. The resulting data export was anonymized.

CroDiab Registry was accessed for research purposes on January 1^st^ 2022. Authors PI and TB had access to personal patient identifiers during data collection. They anonymized the data and then sent it to JD for data analysis. All other authors had access to aggregated data only.

The outcome of SARS-CoV-2 infection was defined as the first or only positive test result (nasopharyngeal swab, Polymerase Chain Reaction (PCR)). According to hospital data, COVID-19 hospitalization outcome was defined as a hospitalization with COVID-19 being the primary or secondary diagnosis. COVID-19 death outcome was defined as death with COVID-19 listed as the primary source of death per the National Causes of Death Registry. The diagnosis of COVID-19 was determined per the World Health Organization International Statistical Classification of Diseases and Related Health Problems, 10th revision (ICD-10), code U07. All COVID-19 diagnoses were laboratory-confirmed by PCR test.

Anti-diabetic drug intake was defined as a prescription that was prescribed at least two times in eight months before the SARS-CoV-2 or COVID-19 outcome. If the person experienced none of the outcomes, therapy was defined if a prescription was picked up at least once eight months before the patient visited her primary healthcare provider with a diagnosis of diabetes mellitus recorded in the system during that visit. Glycated hemoglobin (HbA1C) and body mass index (BMI) data were searched and retrieved six months before outcomes or primary health care visits.

A person is classified as a person with diabetes mellitus if at least one of the following conditions are met: (1) at least one hospital report with diabetes mellitus diagnosis was found in the system, (2) if the person visited their primary healthcare provider at least twice in period of study and ICD-10 diagnosis of E11 was recorded during the visits, (3) if the person was prescribed at least two prescriptions with diagnosis E11 or if the prescriptions had Anatomical Therapeutic Chemical Classification (ATC) codes A10 excluding code A10BA, (4) if person’s primary healthcare provider reported the person as diabetes mellitus patient via the National Public Health Information System plus the person visited her primary healthcare provider at least once and ICD-10 diagnosis of E11 was recorded during the visit or the person was prescribed at least one prescription with diagnosis E11 or if the prescription had ATC codes A10 excluding code A10BA [35].

Chronic kidney disease was defined as ICD-10 diagnosis N18 (Chronic Kidney Disease) and/or E11.21 (Type 2 Diabetes Mellitus with Diabetic Nephropathy) recorded at least twice in the system from Jan 1^st^ 2018 onwards.

Individual comorbidities were identified if their ICD-10 codes were recorded at least twice in the system from Jan 1^st^ 2018 onwards. ICD-10 codes searched for were as follows: malignant neoplasms (C00-C97); hypertensive diseases (I10-I15); ischemic heart diseases (I20-I25); cerebrovascular diseases (I60-I69); diseases of the circulatory system excluding hypertension (I00-I09 and I20-I99); chronic lower respiratory diseases (J40-J47); other chronic obstructive pulmonary disease (J44); cardiomyopathy (I48).

Inclusion criteria for data analysis were type 2 diabetes mellitus, defined as per CroDiab definition already described, and age of 18 years or above. The exclusion criteria were lack of reliable data on anti-diabetic drug use. The latter patients were omitted from the analysis.

### Statistical analysis

Differences between groups of individual conditions were compared with the χ2 test. The level of significance was set at α  =  0.05.

Logistic regression analysis was used to determine the relative risks of developing outcomes. Initially, the model included all available variables. In the final model, selected variables were included. Variables were selected using the backward stepwise Likelihood Ratio (LR) method. In the multiple models, Odds ratios (OR) and 95% Confidence Intervals (CI) were determined.

In our initial statistical analysis plan, these covariates were systematically forced into the models: age, sex, BMI and HbA1c, type 2 diabetes mellitus duration, ACE inhibitors (ACEI) or angiotensin receptor blockers (ARB) intake, SARS-CoV-2 vaccination data, comorbidities, and anti-diabetic drugs. However, models with BMI and HbA1b could not perform due to the deficient number of BMI and HbA1c data available, so these variables were not included in further models.

Analyses were performed with IBM SPSS Statistics software, version 29.0 (IBM, Armonk, NY, USA).

### Ethics

The Croatian Institute of Public Health Ethical Committee (No. 381-15-21-3) and the University of Zagreb, School of Medicine Ethical Committee (No. 641-01/22-02/01) approved the study. The need for informed consent was waived by The Croatian Institute of Public Health Ethical Committee. The study has been performed in accordance with the Declaration of Helsinki.

## Results

There were 310 749 patients with diabetes mellitus type 2 in the CroDiab database older than 18 years of age. After excluding patients without reliable anti-diabetic therapy data (N = 78 953), we were left with 231 796 patients. Out of these, 7 539 had chronic kidney disease (prevalence 3.25%). Table 1 shows the demography and characteristics of these patients.

**Table 1:** Characteristics of patients with diabetes mellitus type 2 and chronic kidney disease (N = 7539).

Demography	Male sex, N (%)	4305 (57.1%)
	Age in years. mean ± SD	73.27±9.35
	Diabetes duration in years. mean ± SD	7.73±4.64
COVID-19 outcomes	SARS-Cov-2 positive, N (%)	1363 (18.1%)
	COVID-19 hospitalized, N (%)	731 (9.7%)
	COVID-19 deaths, N (%)	251 (3.3%)
ACEI	Yes, N (%)	3415 (45.3%)
ARB	Yes, N (%)	617 (8.2%)
SARS-CoV-2 vaccination	Dose 1, N (%)	5039 (66.8%)
	Dose 2, N (%)	4752 (63%)
	Booster, N (%)	2429 (32.2%)
Comorbidities other than chronic kidney disease	Hypertensive diseases, N (%)	7023 (93.2%)
	Diseases of the circulatory system excluding hypertension, N (%)	5741 (76.2%)
	Ischaemic heart diseases, N (%)	2740 (36.3%)
	Cardiomyopathy, N (%)	1742 (23.1%)
	Chronic lower respiratory diseases, N (%)	1477 (19.6%)
	Cerebrovascular diseases, N (%)	1356 (18%)
	Malignant neoplasms, N (%)	1287 (17.1%)
	Other chronic obstructive pulmonary disease, N (%)	1043 (13.8%)
Diabetes mellitus chronic treatment	Biguanides (metformin), N (%)	3016 (40%)
	Sulfonylureas, N (%)	2884 (38.3%)
	DPP-4 inhibitors, N (%)	2463 (32.7%)
	Insulin, N (%)	2004 (26.6%)
	GLP-1 analogues, N (%)	596 (7.9%)
	Meglitinides (repaglinide), N (%)	505 (6.7%)
	SGLT-2 inhibitors, N (%)	447 (5.9%)
	Thiazolidinediones (pioglitazone), N (%)	400 (5.3%)
	Alpha glucosidase inhibitors (acarbose), N (%)	52 (0.7%)

Abbreviations: ACEI = angiotensin-converting enzyme inhibitor. ARB = angiotensin receptor blockers. DPP-4 = Dipeptidyl peptidase 4. SGLT-2 = Sodium-glucose co-transporter 2. GLP-1 = Glucagon-like peptide-1

T2DM-CKD were predominantly male, of old age, with a mean diabetes duration of almost 8 years. Almost all of them have hypertensive diseases and 3/4 have circulatory diseases, 1/3 have ischemic heart disease, 1/4 have cardiomyopathy and 1/4 have a chronic respiratory disease. Forty percent are taking metformin or/and sulfonylurea and 1/3 are taking a DPP-4 inhibitor. One-fourth is treated with insulin. Sixty six percent have received at least one dose of the SARS-CoV-2 vaccine while only about 30% have received a booster. The use of SGLT-2 inhibitors, GLP-1 analogues, pioglitazone, repaglinide and acarbose is below 10%. Two-year cumulative incidence of SARS-CoV-2 infections was 18.1%, COVID-19 hospitalizations 9.7%, and COVID-19 deaths 3.3%.

SARS-CoV-2 and COVID-19 epidemiology in T2DM-CKD is compared with the epidemiology of the diabetic patients without CKD in Table 2.

**Table 2:** SARS-CoV-2 and COVID-19 epidemiology in the studied cohort, comparison to diabetes mellitus type 2 patients without chronic kidney disease.

Outcome	T2DM-CKD (N = 7539)	T2DM no CKD (N = 224 257)	P
SARS-CoV-2 infections	1363 (18.1%)	32378 (14.4%)	<0.001
COVID-19 hospitalizations	731 (9.7%)	9460 (4.2%)	<0.001
COVID-19 deaths, N (%)	251 (3.3%)	2441 (1.1%)	<0.001

Years 2020 and 2021

P values calculated via hi square test

Abbreviations: T2DM-CKD = Patients with diabetes mellitus type 2 and chronic kidney disease; T2DM no CKD = Diabetes mellitus type 2 patients without chronic kidney disease

T2DM-CKD patients have a 20% higher risk of acquiring SARS-CoV-2 infection, more than double the risk of getting hospitalized due to COVID-19, and 3 times the risk of dying from COVID-19 in comparison to the diabetes mellitus type 2patients without CKD.

Table 3 shows outcomes in patient groups according to different antidiabetic drug use.

**Table 3:** SARS-CoV-2/COVID-19 outcomes in patients with diabetes mellitus type 2 and chronic kidney disease depending on antidiabetic therapy.

Antidiabetic medication		COVID-19 outcomes		
		positivity	hospitalization	death
SGLT-2 inhibitor	Yes (N = 414)	**53 (11.9%)**	**16 (3.6%)**	**5 (1.1%)**
	No (N = 6904)	**1310 (18.5%)**	**715 (10.1%)**	**246 (3.5%)**
	p	**<0.001**	**<0.001**	**0.007**
metformin	Yes (N = 2843)	**496 (16.4%)**	**221 (7.3%)**	**83 (2.8%)**
	No (N = 4475)	**867 (19.2%)**	**510 (11.3%)**	**168 (3.7%)**
	p	**0.003**	**<0.001**	**0.022**
sulfonylureas	Yes (N = 2824)	543 (18.8%)	**310 (10.7%)**	100 (3.5%)
	No (N = 4494)	820 (17.6%)	**421 (9%)**	151 (3.2%)
	p	0.184	**0.015**	0.599
DPP-4 inhibitors	Yes (N = 2403)	426 (17.3%)	223 (9.1%)	**68 (2.8%)**
	No (N = 4915)	937 (18.5%)	508 (10%)	**183 (3.6%)**
	p	0.218	0.189	**0.055**
GLP-1 analogues	Yes (N = 565)	105 (17.6%)	**42 (7%)**	14 (2.3%)
	No (N = 6753)	1258 (18.1%)	**689 (9.9%)**	237 (3.4%)
	p	0.76	**0.023**	0.164
acarbose	Yes (N = 48)	9 (17.3%)	5 (9.6%)	3 (5.8%)
	No (N = 7270)	1354 (18.1%)	726 (9.7%)	248 (3.3%)
	p	0.885	0.984	0.325
pioglitazone	Yes (N = 388)	69 (17.3%)	36 (9%)	14 (3.5%)
	No (N = 6930)	1294 (18.1%)	695 (9.7%)	237 (3.3%)
	p	0.658	0.629	0.845
repaglinide	Yes (N = 495)	79 (15.6%)	48 (9.5%)	13 (2.6%)
	No (N = 6823)	1284 (18.3%)	683 (9.7%)	238 (3.4%)
	p	0.141	0.88	0.327
insulin	Yes (N = 1954)	**400 (20%)**	**239 (11.9%)**	**89 (4.4%)**
	No (N = 5364)	**963 (17.4%)**	**492 (8.9%)**	**162 (2.9%)**
	p	**0.011**	**<0.001**	**0.001**

P calculated via hi square test

statistically significant p values are bolded

Patient characteristics for each group are presented in S1 Table

Abbreviations: SGLT-2 = Sodium-glucose Cotransporter-2, DPP-4 = Dipeptidyl Peptidase 4, GLP-1 = Glucagon-like peptide 1

T2DM-CKD taking SGLT-2 inhibitors or metformin had lower incidences of all 3 study outcomes in comparison to patients not taking these drugs. Patients treated with insulin got infected, hospitalized, and died more often than patients not treated with insulin. The sulfonylurea-group had a higher incidence of hospitalizations, while the GLP-1-analogues-group had a lower incidence of hospitalizations. Patients in DPP-4 inhibitors group died less often than patients not taking DPP-4 inhibitors, however P value is of borderline significance (0.055). Patient differences regarding demography, comorbidities, ACEI or ARBs intake, and SARS-CoV-2 vaccination status are presented in S1 Table.

### Results of logistic regression models

#### SARS-CoV-2 positivity

The results of a multivariate regression model for the outcome of positivity are shown in Table 4.

**Table 4:** Multivariate regression model for SARS-CoV-2 positivity, patients with diabetes mellitus type 2 and chronic kidney disease (N = 7539).

Variable	p	Odds Ratio	95% Confidence Interval
**Age in years**	**0.004**	**0.990**	**0.984–0.997**
**Female sex**	**0.001**	**0.814**	**0.719–0.923**
**Diabetes duration in years**	**<0.001**	**1.024**	**1.011–1.037**
**SGLT-2 inhibitors**	**0.001**	**0.607**	**0.448–0.823**
**metformin**	**0.040**	**0.875**	**0.770–0.994**
**repaglinide**	**0.039**	**0.765**	**0.593–0.986**
**SARS-cov-2 vaccination dose 1**	**0.004**	**1.476**	**1.136–1.919**
**SARS-cov-2 vaccination dose 2**	**0.004**	**0.677**	**0.521–0.88**
**SARS-cov-2 vaccination booster**	**<0.001**	**0.276**	**0.231–0.330**
**Chronic lower respiratory diseases**	**0.043**	**1.163**	**1.005–1.347**

Final model, backward stepwise Likelihood Ratio (LR) method; P of the final model <0.001

The bolded text in the table represents variables with statistically significant association with outcome

Abbreviations: SGLT-2 = Sodium-glucose co-transporter 2

Initially, the model included all available variables (variables from Table 1). In the final model, selected variables were included, as presented in Table 4. Variables were selected using the backward stepwise Likelihood Ratio (LR) method.

For SARS-CoV-2 infections, protective factors were older age, female sex, SGLT-2 inhibitors use, metformin use, repaglinide use, and 2^nd^ and 3^rd^ vaccine doses. Duration of diabetes was a risk factor as well as first dose of vaccine and presence of chronic lower respiratory disease.

#### COVID-19 hospitalization

The results of a multivariate regression model for the outcome of hospitalization are shown in Table 5.

**Table 5:** Multivariate regression model for COVID-19 hospitalizations, patients with diabetes mellitus type 2 and chronic kidney disease (N = 7539).

Variable	P	Odds Ratio	95% Confidence Interval
**Female sex**	**<0.001**	**0.71**	**0.604–0.833**
**Insulin**	**<0.001**	**1.411**	**1.167–1.706**
**SGLT-2 inhibitors**	**0.001**	**0.43**	**0.257–0.719**
**Sulfonylureas**	**0.024**	**1.226**	**1.027–1.464**
**Metformin**	**0.004**	**0.769**	**0.643–0.92**
**SARS-cov-2 vaccination dose 2**	**<0.001**	**0.554**	**0.465–0.66**
**SARS-cov-2 vaccination booster**	**<0.001**	**0.275**	**0.209–0.364**
**Diseases of the circulatory system excluding hypertension**	**0.023**	**1.264**	**1.033–1.546**

Final model, backward stepwise Likelihood Ratio (LR) method, P of the final model <0.001

The bolded text in the table represents variables with statistically significant association with outcome

Abbreviations: SGLT-2 = Sodium-glucose co-transporter 2

Initially, the model included all available variables (variables from Table 1). In the final model, selected variables were included, as presented in Table 5. Variables were selected using the backward stepwise Likelihood Ratio (LR) method.

For COVID-19 hospitalizations, female sex, SGLT-2 use, metformin use, and 2^nd^ and 3^rd^ vaccine doses were protective factors. Insulin use, sulfonylurea and presence of diseases of the circulatory system excluding hypertension were risk factors.

#### COVID-19 deaths

The results of a multivariate regression model for the outcome of death are shown in Table 6.

**Table 6:** Multivariate regression model for COVID-19 death, patients with diabetes mellitus type 2 and chronic kidney disease (N = 7539).

Variable	P	Odds Ratio	95% Confidence Interval
**Age in years**	**0.002**	**1.024**	**1.009–1.040**
**Female sex**	**<0.001**	**0.548**	**0.417–0.721**
**Diabetes duration in years**	**<0.001**	**1.049**	**1.020–1.080**
Insulin	0.071	1.314	0.977–1.767
DPP-4 inhibitors	0.067	0.761	0.568–1.019
Repaglinide	0.094	0.610	0.342–1.088
**SARS-cov-2 vaccination dose 2**	**<0.001**	**0.188**	**0.131–0.271**
**SARS-cov-2 vaccination booster**	**<0.001**	**0.073**	**0.022–0.237**

Final model, backward stepwise Likelihood Ratio (LR) method, P of the final model <0.001

The bolded text in the table represents variables with statistically significant association with outcome

Abbreviations: DPP-4 = Dipeptidyl peptidase 4, SGLT-2 = Sodium-glucose co-transporter 2

Initially, the model included all available variables (variables from Table 1). In the final model, selected variables were included, as presented in Table 6. Variables were selected using backward stepwise Likelihood Ratio (LR) method.

For the outcome of COVID-19 death, our models showed protective factors are female sex, and 2^nd^ and 3^rd^ vaccine doses. Risk factors are older age and diabetes duration.

## Discussion

To the best of our knowledge, this is the first paper describing the association of antidiabetic drugs and SARS-CoV-2 and COVID-19 outcomes in T2DM-CKD. This is an important topic because population with both T2DM and CKD is heavily burdened by COVID-19 and because role of antidiabetic drugs in COVID-19 outcomes has been a matter of debate since the beginning of the pandemic with fear they might lead to worse outcomes [5, 18–28].

Our key findings regarding the association between antidiabetic drugs and COVID-19 outcomes in population suffering T2DM and CKD are optimistic for most antidiabetic drugs. No association between antidiabetic drugs and COVID-19 death outcome was found. SGLT-2 inhibitors and metformin were shown to be protective against COVID-19 hospitalization and SARS-CoV-2 infections. Repaglinide also showed protection against the infection. Our study did find insulin and sulfonylurea to be risk factors for COVID-19 hospitalization, though. Nevertheless, all this is far from fears present at the beginning of COVID-19 pandemic.

We identified 7539 T2DM-CKD in our database which makes a prevalence of 3.25% in the population of patients with diabetes mellitus type 2. The prevalence of CKD in the general adult population is 10–15% which means our prevalence estimate is too low [8]. This is probably because diagnosis of CKD is often not recorded in medical records which consequently leads to low numbers of ICD codes N18 (Chronic Kidney Disease) and E11.21 (Type 2 Diabetes Mellitus with Diabetic Nephropathy) in public health databases [8, 36].

When it comes to the use of antidiabetic medications in our T2DM-CKDs, 40% of patients are taking metformin, 38.3% sulfonylureas, 32.7% DPP-4 inhibitors, 26.6% insulin 7.9% GLP-1 analogues, 6.7% repaglinide, 5.9% SGLT-2 inhibitors, 5.3% pioglitazone, 0.7% acarbose. These numbers are lower than Columbian data which describe 17% of T2DM-CKDs taking SGLT-2 inhibitors, 67.3% metformin, and 52.2% DPP-4 inhibitors [37]. GLP-1 analogues use is a bit lower in Columbian study, 5.2% [37]. Our numbers, however, are much higher than the US data—they report initiation rates of 2.5% for SGLT-2 inhibitors, 3.7% for DPP-4 inhibitors, 2.31% for GLP-1 receptor analogues, 4% for insulin, and 5.5% for sulfonylureas in T2DM-CKD [38]. Another study focusing on SGLT-2 inhibitors and GLP-1 analogues prescription in T2DM-CKD noted that 12% and 10% of these patients are taking SGLT-2 inhibitors and GLP-1 analogues, respectively [39].

Novel antidiabetics have renoprotective effects—SGLT-2 and GLP-1 analogues [15, 28]. Therefore, one would expect somewhat higher rates of prescribing these drugs to T2DM-CKD. However, this is not the case, not just in Croatia but also worldwide. Schernthaner et al warned about the “*worldwide inertia to the use of cardiorenal protective glucose-lowering drugs (SGLT2i and GLP-1 RA) in high-risk patients with type 2 diabetes*” [40].

It should be noted that our data refer to years 2018–2021 and the US data years 2007–2019 [38]. This is all prior to SGLT-2 inhibitors gaining regulatory approvals for use in CKD (2021 dapagliflozin, 2023 empagliflozin) [41, 42].

When it comes to renoprotective drugs other than antidiabetics, 45% of our patients are taking an ACEI and 8.3% an ARB, total of 53.3%. In Columbian data, 67.2% of T2DM-CKD are taking ARB and 15.8% ACEI, total of 83% [37]. In the US on the other hand, 17.8% and 56%of T2DM-CKD are initiating ACEI and ARBs, total of 73.8% [38].

We can conclude that the use of renoprotective novel antidiabetics in our cohort is as low as in the rest of the world while our use of ACEI/ARBs is lower than the worldwide trends.

Our data showed T2DM-CKD experienced more SARS-CoV-2 infections, more COVID-19 hospitalizations, and more COVID-19 deaths in the years 2020 and 2021 in comparison to the diabetes mellitus type 2 patients without CKD. This is in line with what we expected [18].

When it comes to the association of antidiabetic drugs and COVID-19, we found no published studies on this patient population with which we could compare our data. We can only comment on the associations of antidiabetic drugs with the general DM2 population.

Our study identified SGLT-2 inhibitors as protective from COVID-19 hospitalization (OR 0.430; 95% CI 0.257–0.719), and SARS-CoV-2 infection (OR 0.607; 95% CI 0.448–0.823). In years 2021 and 2023 SGLT-2 inhibitors dapagliflozin and empagliflozin, respectively, have received EU and the US regulatory approvals for use in CKD since they improve kidney outcomes regardless of their effect on HbA1c levels [41, 42]. The 2022 update of the Kidney Disease Improving Global Outcomes (KDIGO) guidelines for diabetes management in chronic kidney disease recommends the initiation of treatment with an SGLT-2-inhibitor in people with DM type 2 and CKD. By this, SGLT-2-inhibition has been established as a first-line treatment in individuals with DM type 2 and renal disease [43]. This could explain the potential protective effect shown in our cohort.

A meta-analysis and meta-regression conducted specifically to assess SGLT-2 inhibitors in diabetes patients with COVID-19 of a total of 17 studies showed that preadmission use of SGLT-2 inhibitors was associated with reduced mortality and severity of COVID-19. This benefit of SGLT-2 inhibitors on COVID-19 mortality was not significantly affected by patient factors such as age, sex, hypertension, heart failure, HbA1c levels, metformin use, duration of diabetes, and BMI. The paper’s authors suggested SGLT-2 inhibitors could be considered an anti-diabetic drug of choice, especially during the pandemic [44]. Another, Bayesian meta-analysis of 35 studies on several anti-diabetic agents found that SGLT-2 inhibitors could reduce COVID-19 mortality risk in individuals with diabetes [45].

We would like to stress our focus is the use of SGLT-2 inhibitors prior to acquiring COVID-19 infection (pre-admission use) and not use during acute COVID-19 infection since it is known SGLT-2 inhibitors use during acute infection could be related to ketoacidosis [28].

Our study identified repaglinide as potentially protective against SARS-CoV-2 infections (OR0.756; 95% CI 0.593–0.986). We found a non-clinical study that identified repaglinide as one of the potential candidates for the treatment of COVID-19 [46].

Metformin use was identified in our study as protective from COVID-19 hospitalization (OR 0.769; 95% CI 0.643–0.920) and SARS-CoV-2 infection (OR 0.875; 95% CI 0.770–0.994). We found several large meta-analyses regarding metformin’s protective association against COVID-19 death and hospitalization risk [47–49].

Our study did not identify GLP-1 analogues to have any kind of role in COVID-19 outcomes. This comes as a bit of a surprise since GLP-1 analogues have cardiovascular and somewhat renoprotective effects [15]. Some studies have shown protection from COVID-19 mortality in patients with T2DM2 [50]. Recently their use has shown promising effects in reducing excessive inflammation-induced acute lung injury and improving COVID-19 outcomes [51]. In addition to stimulating postprandial insulin secretion, GLP-1 receptor analogues also seem to have beneficial properties such as anti-inflammatory, anti-obesogenic, pulmonary protective effects, and gut microbiome modulating effects [52].

Our study did not identify any association between pioglitazone and acarbose and COVID-19 outcomes. In a meta-analysis, a thiazolidinedione and an alpha-glucosidase inhibitor were also found to be mortality-neutral in patients with diabetes mellitus type 2 and COVID-19 [53].

Our multivariate regression models showed DPP-4 inhibitors were associated with statistically non-significant decrease in odds for COVID-19 death (OR 0.761; 95% CI 0.568–1.019). Some studies have shown DPP-4 inhibitors mortality benefits in diabetic patients with COVID-19 [54]. DPP-4 inhibitors might inhibit entry of the virus, inhibit inflammation and fibrosis [54]. However, several studies have shown negative associations in terms of increased mortality risk and intensive care unit (ICU) admission risk for people who use them [27, 55].

Our data showed use of insulin (OR 1.411; 95% CI 1.167–1.706) and sulfonylurea (OR 1.226; 95% CI 1.027–1.464) could be related to higher odds of COVID-19 hospitalizations. This is in line with the DM2 population data [23, 45, 47]. When it comes to insulin, it should be noted that in all cited meta-analyses insulin was shown to be independent predictor of COVID-19 mortality or hospitalization regardless of the age, sex, diabetes duration and gylcemic status [23, 45, 47]. The underlying mechanism for insulin’s association with COVID-19 hospitalization and death outcomes is unclear [47].

### Study limitations

Our study has several strengths and several limitations. We described the entire population of T2DM-CKD of the Republic of Croatia and not just a sample. Also, we provided information regarding other comorbidities that could affect COVID-19 outcomes, such as chronic obstructive pulmonary disease which is often not the case in before mentioned studies published in DM2 population.

The limitations of the study are retrospective and observational design. Further on, part of the population was excluded from the analysis due to no medication data. Also, HbA1c, and BMI data, could not be included in logistic regression models due to insufficient data, as well as CKD stage data we currently do not have access to. Also, we currently do not have access to albuminuria or glomerular filtration (GF) data and therefore we have missed to identify the patients who have kidney damage that does not qualify as ICD diagnosis N18 or E11.21. Not having access to patients’ medical records and missing clinical data such as BMI, HbA1c, albuminuria, GF is a known drawback of working with public health databases [56].

Low availability of HbA1c and BMI data can to a certain extent be explained by the COVID-19 pandemic which has had a negative effect on the utilization of healthcare by diabetes patients with decreased numbers of diabetes panels (one of the sources of HbA1c and BMI data) and decreased numbers of visits to primary healthcare providers for diabetes-related problems and diabetes patients who visited their primary healthcare provider [57].

The accuracy of the coding of ICD diagnoses is another issue inherent to working with public health databases. Since we do not have access to medical records, checking the coding manually would be out of the scope of this study.

Additionally, analyzed data were collected during 2020 and 2021, when the original SARS-CoV-2 was still dominant. Therefore our analysis results may not be applied to other SARS-CoV-2 variants.

### Conclusion

In conclusion, the relationship between the use of antidiabetic drugs and COVID-19 outcomes is important in clinical practice and to policymakers because of the growing prevalence of both diabetes mellitus and CKD, the increasing use of glucose-lowering drugs with renoprotective effect, the higher COVID-19 mortality observed in patients with T2DM-CKD and the unpredictable waves of COVID-19 despite vaccines [15, 26]. Drugs such as SGLT-2 inhibitors, metformin, and repaglinide seem not only to be safe in the age of COVID-19 but also beneficial.

Further research in this patient population is needed. It would be beneficial to asses association between antidiabetic drugs combinations and COVID-19 outcomes as well as between combinations of antidiabetic medications with ACEIs/ARBs and statins and COVID-19 outcomes. Further on, constant improvements in the field of data availability in public health databases are necessary.

## Supporting information

S1 Table SARS-CoV-2 and COVID-19 outcomes and patient characteristics in groups depending on antidiabetic therapy. (XLSX)

## References

[1] Martín Sánchez FJ, Martínez-Sellés M, Molero García JM, Moreno Guillén S, Rodríguez-Artalejo FJ, Ruiz-Galiana J, et al. Insights for COVID-19 in 2023. Rev Esp Quimioter. 2023;36(2):114–124. doi: 10.37201/req/122.2022 Epub 2022 Dec 13. ; PMCID: PMC10066911. 36510683 PMC10066911

[2] World Health Organization. With the international public health emergency ending, WHO/Europe launches its transition plan for COVID-19 [internet]. 12 June 2023 [cited October 2023]. Available at: https://www.who.int/europe/news/item/12-06-2023-with-the-international-public-health-emergency-ending—who-europe-launches-its-transition-plan-for-covid-19

[3] Lancet The. The COVID-19 pandemic in 2023: far from over. Lancet. 2023;401(10371):79. doi: 10.1016/S0140-6736(23)00050-8 Erratum in: Lancet. 2023;401(10374):346. . 36641201

[4] Ceriello A, Schnell O. COVID-19: Considerations of Diabetes and Cardiovascular Disease Management. J Diabetes Sci Technol. 2020;14(4):723–4 doi: 10.1177/1932296820930025 Epub 2020 May 30. ; PMCID: PMC7673148. 32475168 PMC7673148

[5] Ceriello A, Catrinoiu D, Chandramouli C, Cosentino F, Dombrowsky AC, Itzhak B, et al. Heart failure in type 2 diabetes: current perspectives on screening, diagnosis and management. Cardiovasc Diabetol. 2021;20(1):218 doi: 10.1186/s12933-021-01408-1 ; PMCID: PMC8571004. 34740359 PMC8571004

[6] Vergara P, Rossi L, Biagi A, Falasconi G, Pannone L, Zanni A, et al. Role of comorbidities on the mortality in patients with SARS-CoV-2 infection: an Italian cohort study. Minerva Med. 2023;114(2):185–190. doi: 10.23736/S0026-4806.21.07187-1 Epub 2021 Apr 29. . 33913658

[7] Gupta A, Marzook H, Ahmad F. Comorbidities and clinical complications associated with SARS-CoV-2 infection: an overview. Clin Exp Med. 2023;23(2):313–331. doi: 10.1007/s10238-022-00821-4 Epub 2022 Apr 1. ; PMCID: PMC8972750. 35362771 PMC8972750

[8] European Renal Association–European Dialysis and Transplantation Association (ERA-EDTA Council); ERACODA Working Group. Chronic kidney disease is a key risk factor for severe COVID-19: a call to action by the ERA-EDTA. Nephrol Dial Transplant. 2021;36(1):87–94. doi: 10.1093/ndt/gfaa314 ; PMCID: PMC7771976. 33340043 PMC7771976

[9] Clark A, Jit M, Warren-Gash C, Guthrie B, Wang HHX, Mercer SW, et al. Global, regional, and national estimates of the population at increased risk of severe COVID-19 due to underlying health conditions in 2020: a modelling study. Lancet Glob Health 2020; 8: e1003–e1017 doi: 10.1016/S2214-109X(20)30264-3 Epub 2020 Jun 15. ; PMCID: PMC7295519. 32553130 PMC7295519

[10] Emami A, Javanmardi F, Pirbonyeh N, Akbari A. Prevalence of Underlying Diseases in Hospitalized Patients with COVID-19: a Systematic Review and Meta-Analysis. Arch Acad Emerg Med. 2020;8(1):e35. ; PMCID: PMC7096724. 32232218 PMC7096724

[11] Henry BM, Lippi G. Chronic kidney disease is associated with severe coronavirus disease 2019 (COVID-19) infection. Int Urol Nephrol. 2020;52(6):1193–4. doi: 10.1007/s11255-020-02451-9 Epub 2020 Mar 28. ; PMCID: PMC7103107. 32222883 PMC7103107

[12] Extance A. Covid-19 and long term conditions: what if you have cancer, diabetes, or chronic kidney disease? BMJ. 2020;368:m1174. doi: 10.1136/bmj.m1174 Erratum in: BMJ. 2020 Mar 27;368:m1270. . 32213482

[13] Lai S, Gigante A, Pellicano C, Mariani I, Iannazzo F, Concistrè A, et al. Kidney dysfunction is associated with adverse outcomes in internal medicine COVID-19 hospitalized patients. Eur Rev Med Pharmacol Sci. 2023;27(6):2706–2714. doi: 10.26355/eurrev_202303_31809 . 37013790

[14] Girgin S, Aksun M, Tüzen AS, Şencan A, Şanlı O, Kırbaş G, et al. Effects of comorbidities associated with COVID-19 cases in Intensive Care Unit on mortality and disease progression. Eur Rev Med Pharmacol Sci. 2023;27(8):3753–3765. doi: 10.26355/eurrev_202304_32174 37140324

[15] Russell CD, Lone NI, Baillie JK. Comorbidities, multimorbidity and COVID-19. Nat Med. 2023;29(2):334–343. doi: 10.1038/s41591-022-02156-9 Epub 2023 Feb 16. . 36797482

[16] Gibertoni D, Reno C, Rucci P, et al. COVID-19 incidence and mortality in non-dialysis chronic kidney disease patients. PLoS ONE. 2021;16:e0254525. doi: 10.1371/journal.pone.0254525 34242368 PMC8270438

[17] Agrawal U, Azcoaga-Lorenzo A, Fagbamigbe AF, Vasileiou E, Henery P, Simpson CR, et al. Association between multimorbidity and mortality in a cohort of patients admitted to hospital with COVID-19 in Scotland. J R Soc Med. 2022;115(1):22–30. doi: 10.1177/01410768211051715 Epub 2021 Oct 21. ; PMCID: PMC8811325. 34672832 PMC8811325

[18] Chudasama YV, Zaccardi F, Gillies CL, Razieh C, Yates T, Kloecker DE, et al. Patterns of multimorbidity and risk of severe SARS-CoV-2 infection: an observational study in the U.K. BMC Infect Dis. 2021;21(1):908. doi: 10.1186/s12879-021-06600-y ; PMCID: PMC8418288. 34481456 PMC8418288

[19] Cai X, Liu X, Sun L, He Y, Zheng S, Zhang Y, et al. Prediabetes and the risk of heart failure: A meta-analysis. Diabetes Obes Metab. 2021;23(8):1746–53. doi: 10.1111/dom.14388 Epub 2021 Apr 8. . 33769672

[20] Bornstein SR, Rubino F, Khunti K, Mingrone G, Hopkins D, Birkenfeld AL, et al. Practical recommendations for the management of diabetes in patients with COVID-19. Lancet Diabetes Endocrinol. 2020;8(6):546–550. doi: 10.1016/S2213-8587(20)30152-2 Epub 2020 Apr 23. ; PMCID: PMC7180013. 32334646 PMC7180013

[21] Chen CF, Chen YT, Chen TH, Chen FY, Yang YP, Wang ML, et al. Judicious use of sodium-glucose cotransporter 2 inhibitors in patients with diabetes on coronavirus-19 pandemic. J Chin Med Assoc. 2020;83(9):809–811. doi: 10.1097/JCMA.0000000000000354 ; PMCID: PMC7434023. 32433344 PMC7434023

[22] Ceriello A, Stoian AP, Rizzo M. COVID-19 and diabetes management: What should be considered? Diabetes Res Clin Pract. 2020;163:108151. doi: 10.1016/j.diabres.2020.108151 Epub 2020 Apr 17. ; PMCID: PMC7162752. 32305399 PMC7162752

[23] Katsiki N, Ferrannini E. Anti-inflammatory properties of antidiabetic drugs: A "promised land" in the COVID-19 era? J Diabetes Complications. 2020;34(12):107723. doi: 10.1016/j.jdiacomp.2020.107723 Epub 2020 Aug 26. ; PMCID: PMC7448766. 32900588 PMC7448766

[24] Banerjee Y, Pantea Stoian A, Silva-Nunes J, Sonmez A, Rizvi AA, Janez A, et al. The role of GLP-1 receptor agonists during COVID-19 pandemia: a hypothetical molecular mechanism. Expert Opin Drug Saf. 2021;20(11):1309–1315. doi: 10.1080/14740338.2021.1970744 Epub 2021 Aug 25. ; PMCID: PMC8425441. 34424130 PMC8425441

[25] Salmen T, Pietroșel VA, Mihai BM, Bica IC, Teodorescu C, Păunescu H, et al. Non-Insulin Novel Antidiabetic Drugs Mechanisms in the Pathogenesis of COVID-19. Biomedicines. 2022;10(10):2624. doi: 10.3390/biomedicines10102624 ; PMCID: PMC9599217. 36289885 PMC9599217

[26] Ferrannini G, Lund LH, Benson L, Rizzo M, Almahmeed W, Rosano GMC, et al. Association between use of novel glucose-lowering drugs and COVID-19 hospitalization and death in patients with type 2 diabetes: a nationwide registry analysis. Eur Heart J Cardiovasc Pharmacother. 2022;9(1):10–17. doi: 10.1093/ehjcvp/pvac044 Erratum in: Eur Heart J Cardiovasc Pharmacother. 2023 Jan 31: ; PMCID: PMC9384777. 35963647 PMC9384777

[27] Dalan R, Ang LW, Tan WYT, Fong SW, Tay WC, Chan YH, et al. The association of hypertension and diabetes pharmacotherapy with COVID-19 severity and immune signatures: an observational study. Eur Heart J Cardiovasc Pharmacother. 2021;7(3):e48–e51. doi: 10.1093/ehjcvp/pvaa098 ; PMCID: PMC7454507. 32766831 PMC7454507

[28] Khunti K, Aroda VR, Bhatt DL, Bozkurt B, Buse JB, Heerspink HL, et al. Re-examining the widespread policy of stopping sodium-glucose cotransporter-2 inhibitors during acute illness: A perspective based on the updated evidence. Diabetes Obes Metab. 2022;24(11):2071–2080. doi: 10.1111/dom.14805 Epub 2022 Aug 4. . 35801339

[29] Poljicanin T, Bralic Lang V, Mach Z, Svajda M. Croatian diabetes registry (CroDiab) and implementation of standardised diabetes checklists using Joint Action CHRODIS Recommendations and Criteria. Ann Ist Super Sanita. 2021;57(1):74–9 doi: 10.4415/ANN_21_01_12 33797409

[30] Poljicanin T, Pavlić-Renar I, Metelko Z. CroDiab NET—registar osoba sa šećernom bolesti [CroDiab NET—electronic diabetes registry]. Acta Med Croatica. 2005;59(3):185–9 [Croatian] 16095190

[31] Poljičanin T, Pristaš I. National public health information system in Croatia. International Public Health Conference "Health indicators as an important tool for strengthening health information systems in the European Region" Tirana, Albania, 2016

[32] Koncar M, Gvozdanović D. Primary healthcare information system—the cornerstone for the next generation healthcare sector in Republic of Croatia. Int J Med Inform. 2006;75(3–4):306–14. doi: 10.1016/j.ijmedinf.2005.08.007 Epub 2005 Oct 4. . 16213189

[33] Capak K, Kopal R, Benjak T, Cerovečki I, Draušnik Ž, Bucić L, et al. Surveillance system for coronavirus disease 2019 epidemiological parameters in Croatia. Croat Med J. 2020;61(6):481–482. doi: 10.3325/cmj.2020.61.481 ; PMCID: PMC7821359. 33410292 PMC7821359

[34] Antoljak N, Erceg M. Značaj digitalizacije podataka prijave uzroka smrti za unapređenje kvalitete epidemiološkog nadzora [Role of digitalized death reports in epidemiological surveillance quality] Med. inform. 2021;15:95 [Croatian]

[35] Croatian Institute of Public Health (CIPH). National Registry of Patients with Diabetes (CroDiab). Report for year 2020. [internet]. Available at: https://www.hzjz.hr/wp-content/uploads/2021/05/Izvje%C5%A1%C4%87e-za-2020.-godinu.pdf Accessed 26 March 2023

[36] Perez-Gomez MV, Bartsch LA, Castillo-Rodriguez E, Fernandez-Prado R, Fernandez-Fernandez B, Martin-Cleary C, et al. Clarifying the concept of chronic kidney disease for non-nephrologists. Clin Kidney J. 2019 Feb 14;12(2):258–61. doi: 10.1093/ckj/sfz007 ; PMCID: PMC6452188. 30976406 PMC6452188

[37] Machado-Duque ME, Gaviria-Mendoza A, Valladales-Restrepo LF, Franco JS, de Rosario Forero M, Vizcaya D, et al. Treatment patterns of antidiabetic and kidney protective therapies among patients with type 2 diabetes mellitus and chronic kidney disease in Colombia. The KDICO descriptive study. Diabetol Metab Syndr. 2023;15(1):150. doi: 10.1186/s13098-023-01126-6 ; PMCID: PMC10318702 37403118 PMC10318702

[38] Fried L, Schmedt N, Folkerts K, Bowrin K, Raad H, Batech M, et al. High unmet treatment needs in patients with chronic kidney disease and type 2 diabetes: real-world evidence from a US claims database. Nephrol Dial Transplant. 2023;38(3):630–643. doi: 10.1093/ndt/gfac140 ; PMCID: PMC9976755. 35389468 PMC9976755

[39] Lamprea-Montealegre JA, Madden E, Tummalapalli SL, Chu CD, Peralta CA, Du Y, et al. Prescription Patterns of Cardiovascular- and Kidney-Protective Therapies Among Patients With Type 2 Diabetes and Chronic Kidney Disease. Diabetes Care. 2022;45(12):2900–2906. doi: 10.2337/dc22-0614 ; PMCID: PMC9998844 36156061 PMC9998844

[40] Schernthaner G, Shehadeh N, Ametov AS, Bazarova AV, Ebrahimi F, Fasching P, et al. Worldwide inertia to the use of cardiorenal protective glucose-lowering drugs (SGLT2i and GLP-1 RA) in high-risk patients with type 2 diabetes. Cardiovasc Diabetol. 2020;19(1):185. doi: 10.1186/s12933-020-01154-w ; PMCID: PMC7585305 33097060 PMC7585305

[41] AstraZeneca 2021. Forxiga approved in the EU for the treatment of chronic kidney disease in patients with and without type-2 diabetes [internet]. Available at: https://www.astrazeneca.com/media-centre/press-releases/2021/forxiga-approved-in-the-eu-for-ckd.html

[42] Boehringer Ingelheim 2023. Jardiance® (empagliflozin) approved in the EU for the treatment of adults with chronic kidney disease [internet]. Available at: https://www.boehringer-ingelheim.com/new-treatment-for-chronic-kidney-disease-approved-in-the-eu

[43] Gerdes C, Müller N, Wolf G, Busch M. Nephroprotective Properties of Antidiabetic Drugs. J Clin Med. 2023;12(10):3377. doi: 10.3390/jcm12103377 ; PMCID: PMC10219007 37240483 PMC10219007

[44] Permana H, Audi Yanto T, Ivan Hariyanto T. Pre-admission use of sodium glucose transporter-2 inhibitor (SGLT-2i) may significantly improves Covid-19 outcomes in patients with diabetes: A systematic review, meta-analysis, and meta-regression. Diabetes Res Clin Pract. 2023;195:110205. doi: 10.1016/j.diabres.2022.110205 Epub 2022 Dec 9. ; PMCID: PMC9731816. 36502891 PMC9731816

[45] Chen Y, Lv X, Lin S, Arshad M, Dai M. The Association Between Antidiabetic Agents and Clinical Outcomes of COVID-19 Patients With Diabetes: A Bayesian Network Meta-Analysis. Front Endocrinol (Lausanne). 2022;13:895458. doi: 10.3389/fendo.2022.895458 ; PMCID: PMC9186017. 35692410 PMC9186017

[46] Qu H, Zheng Y, Wang Y, Li H, Liu X, Xiong X, et al. The potential effects of clinical antidiabetic agents on SARS-CoV-2. J Diabetes. 2021;13(3):243–252. doi: 10.1111/1753-0407.13135 Epub 2020 Dec 19. ; PMCID: PMC7753367 33210826 PMC7753367

[47] Kan C, Zhang Y, Han F, Xu Q, Ye T, Hou N, et al. Mortality Risk of Antidiabetic Agents for Type 2 Diabetes With COVID-19: A Systematic Review and Meta-Analysis. Front Endocrinol (Lausanne). 2021;12:708494. doi: 10.3389/fendo.2021.708494 ; PMCID: PMC8481667. 34603199 PMC8481667

[48] Nassar M, Abosheaishaa H, Singh AK, Misra A, Bloomgarden Z. Noninsulin-based antihyperglycemic medications in patients with diabetes and COVID-19: A systematic review and meta-analysis. J Diabetes. 2023;15(2):86–96. doi: 10.1111/1753-0407.13359 Epub 2023 Jan 23. ; PMCID: PMC9934962. 36690377 PMC9934962

[49] Ganesh A, Randall MD. Does metformin affect outcomes in COVID-19 patients with new or pre-existing diabetes mellitus? A systematic review and meta-analysis. Br J Clin Pharmacol. 2022;88(6):2642–2656. doi: 10.1111/bcp.15258 Epub 2022 Feb 23. ; PMCID: PMC9111510. 35122284 PMC9111510

[50] Hariyanto TI, Intan D, Hananto JE, Putri C, Kurniawan A. Pre-admission glucagon-like peptide-1 receptor agonist (GLP-1RA) and mortality from coronavirus disease 2019 (Covid-19): A systematic review, meta-analysis, and meta-regression. Diabetes Res Clin Pract. 2021;179:109031. doi: 10.1016/j.diabres.2021.109031 Epub 2021 Aug 28. ; PMCID: PMC8397482. 34461139 PMC8397482

[51] Pang J, Liu M, Ling W, Jin T. Friend or foe? ACE2 inhibitors and GLP-1R agonists in COVID-19 treatment. Obes Med. 2021;22:100312. doi: 10.1016/j.obmed.2020.100312 Epub 2021 Jan 6. ; PMCID: PMC7785422. 33426364 PMC7785422

[52] Belančić A, Kresović A, Troskot Dijan M. Glucagon-like peptide-1 receptor agonists in the era of COVID-19: Friend or foe? Clin Obes. 2021;11(2):e12439. doi: 10.1111/cob.12439 Epub 2021 Jan 10. ; PMCID: PMC7995087. 33423388 PMC7995087

[53] Nguyen NN, Ho DS, Nguyen HS, Ho DKN, Li HY, Lin CY, et al. Preadmission use of antidiabetic medications and mortality among patients with COVID-19 having type 2 diabetes: A meta-analysis. Metabolism. 2022;131:155196. doi: 10.1016/j.metabol.2022.155196 Epub 2022 Mar 31. ; PMCID: PMC8970613. 35367460 PMC8970613

[54] Narayanan N, Naik D, Sahoo J, Kamalanathan S. Dipeptidyl peptidase 4 inhibitors in COVID-19: Beyond glycemic control. World J Virol. 2022;11(6):399–410. doi: 10.5501/wjv.v11.i6.399 ; PMCID: PMC9724202. 36483108 PMC9724202

[55] Khunti K, Knighton P, Zaccardi F, Bakhai C, Barron E, Holman N, et al. Prescription of glucose-lowering therapies and risk of COVID-19 mortality in people with type 2 diabetes: a nationwide observational study in England. Lancet Diabetes Endocrinol. 2021;9(5):293–303. doi: 10.1016/S2213-8587(21)00050-4 Epub 2021 Mar 30. ; PMCID: PMC8009618. 33798464 PMC8009618

[56] Cunningham SG, Carinci F, Brillante M, Leese GP, McAlpine RR, Azzopardi J, et al Core Standards of the EUBIROD Project. Defining a European Diabetes Data Dictionary for Clinical Audit and Healthcare Delivery. Methods Inf Med. 2016;55(2):166–76 10.3414/ME15-01-0016 26666452

[57] Cerovečki I, Švajda M. COVID-19 Pandemic Influence on Diabetes Management in Croatia. Front Clin Diabetes Healthc. 2021 Mar 21;2:704807 doi: 10.3389/fcdhc.2021.704807 36994328 PMC10012086

